# Evaluación del proceso de teleconsulta desde la perspectiva del proveedor, Programa de Telesalud de Oaxaca, México

**DOI:** 10.26633/RPSP.2017.22

**Published:** 2017-04-21

**Authors:** Mauricio Velázquez, Adrián Pacheco, Miriam Silva, y Dámaris Sosa

**Affiliations:** 1 Centro Nacional de Tecnología en Salud Secretaría de Salud México D.F. México Centro Nacional de Tecnología en Salud, Secretaría de Salud, México D.F., México.

**Keywords:** Telemedicina, consulta remota, México, Telemedicine, remote consultation, Mexico

## Abstract

**Objective:**

Identify barriers to implementation of the teleconsultation process in order to develop strategies to improve the program’s operation.

**Methods:**

A process evaluation strategy was used to study the implementation of the teleconsultation service. The program’s operating manuals were compared with the qualitative and quantitative information compiled on the practical implementation of the teleconsultation process.

**Results:**

The factors reported as obstacles to the teleconsultation process were: slow Internet connection, the hours available to the public, the specialized services offered, and insufficient clinical history included in teleconsultation requests. It was determined that 60% of internal medicine patients received two or more teleconsultations in the study period, as did 44% of patients of the gynecology service. Four consulting medical units accounted for 75% of the teleconsultations and the rest were distributed among 12 medical units.

**Conclusions:**

The barriers identified in the teleconsultation process mainly affect consulting physicians; even so, productivity is on an upward trend. Despite the existing barriers, it was determined that some patients receive follow-up through the program, which favors access to care. It is necessary to standardize implementation and to conduct subsequent research on patients’ health condition.

## RESUMEN

**Objetivo.:**

*Identificar las barreras en la implementación relacionadas con el proceso de teleconsulta para desarrollar estrategias que mejoren la operación del programa*.

**Métodos.:**

*Se estudió la implementación del servicio de teleconsulta mediante una estrategia de evaluación de procesos. Los referentes fueron los manuales operativos del programa, los cuales fueron comparados con información qualitativa y cuantitativa de la práctica del proceso de teleconsulta*.

**Resultados.:**

*Los factores reportados como obstáculos en el proceso de teleconsultas fueron la lentitud de la conexión a internet, el horario de atención, la oferta de especialidades y la insuficiencia de antecedentes clínicos en la solicitud de teleconsulta. Se identificó que 60% de los pacientes del servicio de medicina interna recibieron dos o más teleconsultas en el período de estudio, así como 44% de las pacientes del servicio de ginecología. Cuatro unidades médicas consultantes concentraron 75% de las teleconsultas, el resto se distribuyó en 12 unidades médicas*.

**Conclusiones.:**

*Las barreras identificadas en el proceso de teleconsulta afectan principalmente al médico consultante; aun así, la productividad se halla en aumento. Pese a las barreras existentes, se identificó que hay pacientes que reciben seguimiento por parte del programa, lo que favorece el acceso a la atención. Se requiere fortalecer la implementación y realizar investigaciones posteriores sobre las condiciones de salud de los pacientes*.

En la última década, diversas herramientas de apoyo y coordinación para el impulso a las estrategias nacionales de telesalud han sido organizadas por la Organización Mundial de la Salud (OMS), la Organización Panamericana de la Salud (OPS), el Banco Interamericano de Desarrollo (BID) y la Comisión Económica para América Latina y el Caribe (CEPAL) (1–4).

Las estrategias nacionales de telesalud en América Latina han aumentado su número a partir del año 2000. México y Costa Rica han iniciado estos proyectos en los años 1995 y 1996, respectivamente; en los siguientes años emergieron las estrategias de Panamá (2002), Ecuador (2006), Colombia (2007), Brasil (2007), Perú (2007), El Salvador (2010), Guatemala y Venezuela (2012) y, recientemente, Bolivia (1).

Los objetivos de estas estrategias se orientan a reducir las inequidades en el acceso a la atención médica, complementar la atención primaria y favorecer el alcance de la atención de especialidad. Las zonas geográficas de influencia de la telesalud son áreas excluidas, dispersas o de difícil acceso. Las experiencias en telesalud tienen una estrecha relación con las estrategias de digitalización y fortalecimiento de redes de atención impulsadas por la OPS (1, 5, 6).

Aunque existen diversas definiciones de telesalud, todas tienen un elemento en común: el uso de tecnologías de información y comunicación para prestar servicios médicos, proporcionar educación y en aplicaciones administrativas como los expedientes clínicos electrónicos (2, 7, 8).

## Antecedentes de telesalud en México

La estructura del sistema de salud en México comprende dos sectores, el público y el privado; para los objetivos del artículo se describe a grandes rasgos solo el sector público y sus beneficiarios (9).

El sector público está dividido en dos grandes grupos: 1) las instituciones de seguridad social, las cuales proporcionan atención los trabajadores asalariados, los jubilados y sus familias (Instituto Mexicano del Seguro Social [IMSS], Instituto de Seguridad y Servicios Sociales de los Trabajadores del Estado [ISSSTE] y los servicios de las fuerzas armadas), y 2) las instituciones y programas que atienden a la población sin seguridad social, formado por la Secretaría de Salud a nivel federal; los Servicios Estatales de Salud (SESA), los cuales reciben financiamiento por parte del Seguro Popular de Salud a nivel subnacional; y el Programa IMSS-Prospera. Este segundo grupo atiende a los autoempleados, trabajadores del sector informal, desempleados y personas que se encuentran fuera del mercado de trabajo y a sus familias (9).

En 2010, en los hospitales de la Secretaría de Salud y los SESA, 46 648 médicos estuvieron en contacto con el paciente (10). Esto representa una relación de 1,1 médicos por cada mil habitantes sin seguridad social, cifra por debajo del estándar de tres médicos por mil habitantes recomendado por la Organización Mundial de la Salud (OMS) (11). Con esta restricción, los SESA enfrentan el desafío de fortalecer la oferta de servicios en zonas marginadas y de alta dispersión poblacional.

A fines de 2015, los servicios de telemedicina en el sector público en México estaban presentes en 671 unidades médicas, 450 de ellas en los SESA. Estas unidades están distribuidas en 15 programas estatales de salud que cuentan con el servicio de teleconsulta por videoconferencia (12).

En el mismo período, estos programas registraron una productividad de 45 000 teleconsultas, en las especialidades psiquiatría (31%), medicina interna (25%), ginecología (11%), pediatría (6%), dermatología (6%), cirugía (5%) y otras especialidades (17%). Durante 2015, el Programa de Telesalud de Oaxaca contribuyó con 11% del total de teleconsultas otorgadas a nivel nacional, por debajo de Yucatán (71%), Tamaulipas (29%), San Luis Potosí (23%) y Nuevo León (23%) (13).

En 2014, los SESA de Oaxaca, un estado ubicado en el suroeste de México, contaban con 19 hospitales básicos comunitarios (con capacidad para 12 a 18 camas). En 17 de ellos, la plantilla de médicos no disponía de especialistas en medicina interna; de igual forma, seis de estos hospitales no contaban con médicos ginecológos (14).

El Programa de Telesalud de Oaxaca proporciona teleconsultas a una población potencial de 190 100 personas (12) que se encuentran en la zona de influencia de unidades médicas que conforman la Red de Telesalud de Oaxaca. La Red cuenta con 19 unidades consultantes, diez de ellas son hospitales básicos comunitarios2.

La Coordinación del Programa se encuentra en una unidad médica en la que laboran los médicos especialistas que proporcionan interconsultas mediante el uso de tecnologías de información y comunicación con médicos generales en zonas remotas. En este escenario, las teleconsultas son una modalidad síncrona de la telemedicina. En ellas, el médico consultante y el paciente establecen comunicación por videoconferencia con el médico interconsultante en tiempo real (15). Esta unidad se aloja en un inmueble dedicado solo a la atención médica a distancia. Cuenta con siete médicos interconsultantes: seis médicos especialistas (cirugía, ginecología, radiología, medicina interna y pediatría) y un psicólogo que laboran de lunes a viernes en turno matutino, excepto por el servicio de medicina interna, que tiene horario matutino y vespertino.

En un reporte de casos sobre el servicio de medicina interna del Programa de Telesalud de Oaxaca, se recopiló información de las valoraciones preoperatorias realizadas a 142 pacientes durante los años 2009 a 2011 (16). A los pacientes se les realizaron valoraciones preoperatorias para cirugías electivas mediante teleconsultas. El plazo máximo en el que se realizaron las valoraciones fue de 15 días naturales. Asimismo, se estimó que la atención por telemedicina ocasionó un ahorro estimado de 4 629 pesos mexicanos en promedio por paciente. En estos casos, el Programa proporcionó atención en un período de dos semanas en comparación con 12,8 semanas reportadas en los SESA (17).

**FIGURA 1.**
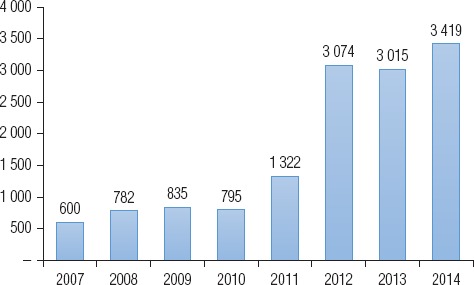
Evolución de teleconsultas proporcionadas por el Programa de Telesalud de Oaxaca, 2007–2014

La evolución del número de teleconsultas del Programa en Oaxaca muestra dos etapas en su implementación. Entre el año 2007 y el 2010, el número de consultas fue inferior a mil teleconsultas anuales, mientras que en el período 2011–2014 se proporcionaron alrededor de 3 000 teleconsultas por año (figura 1). Se desconocen los motivos por los que ha aumentado el número de consultas en este período. Sin embargo, cabe preguntarse cuáles son los factores que permitirían incrementar la cobertura del Programa en los próximos años.

***Fuente:*** Elaborado con información de la Coordinación de Telesalud Oaxaca a noviembre de 2014.

***Nota:*** los datos de 2014 son al 25 de octubre de 2014 con base en información del *software* de Registro Clínico de Información del Programa.

A partir de esta pregunta, se identificó que una evaluación formativa o de procesos es un enfoque pertinente de investigación, ya que tiene el potencial para contribuir directamente a la mejora de un programa, aportando observaciones y recomendaciones acerca de la forma en que el programa opera en la práctica clínica (18, 19).

## OBJETIVO

El objetivo de este trabajo fue realizar un análisis de la gestión operativa del proceso de teleconsultas para valorar si cumple con lo necesario para el logro de los objetivos del Programa de Telesalud de Oaxaca y emitir recomendaciones que permitan instrumentar mejoras.

## Objetivos específicos

- Describir la operación del proceso de teleconsulta desde el punto de vista del prestador de servicios de salud.
- Identificar y analizar los problemas o limitantes, tanto normativos como operativos, internos y externos al programa, que obstaculizan el proceso de teleconsulta.
- Identificar fortalezas y debilidades de la gestión para emitir recomendaciones factibles de implementar tanto a nivel normativo como operativo.

## MÉTODOS

La presente investigación forma parte de la iniciativa denominada “Mejorando la ejecución de programas mediante investigación integrada en la implementación” (iPIER, por sus siglas en inglés). Esta iniciativa es desarrollada por la Alianza para la Investigación en Políticas y Sistemas de Salud (AHPSR, por sus siglas en inglés), en colaboración con la OPS.

**FIGURA 2.**
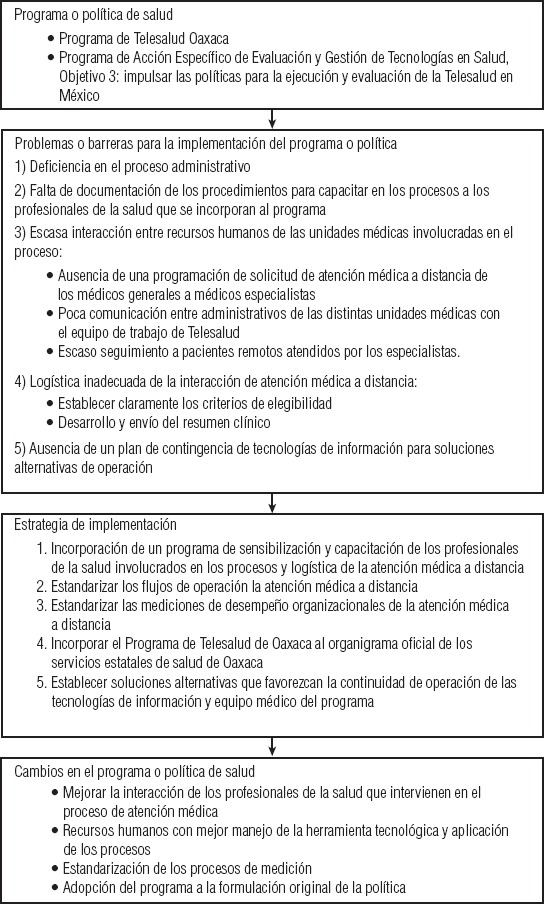
Flujograma del protocolo de investigación iPier

El modelo iPIER coloca a los ejecutores de programas en el centro de la investigación con el objetivo de entender las fallas en los sistemas de salud que crean barreras a la implementación, así como identificar soluciones a estas barreras (figura 2). La investigación sobre la implementación de programas y políticas de salud permite esclarecer el funcionamiento real de dichas iniciativas. Específicamente, la investigación sobre los procesos de implementación promueve la generación de evidencias para impulsar buenas prácticas clínicas y de salud pública. Una descripción detallada de la metodología de investigación integrada en la implementación se incluye en el documento conceptual iPIER (20).

La metodología de evaluación de procesos combina elementos cualitativos y cuantitativos para agregar explicaciones sobre el contexto en que se desarrollan las intervenciones. En general, este tipo de evaluación consiste en el análisis de gabinete de fuentes de información secundarias y la recopilación de información primaria mediante diseño de instrumentos y trabajo de campo (18, 21, 22).

## Plan de recopilación de información y análisis

El trabajo de campo comenzó con una entrevista inicial al coordinador del Programa de Telesalud de Oaxaca, a quien se le consultó sobre posibles actores clave a quienes se pudiera entrevistar para indagar sobre cómo institucionalizar el Programa. Esto permitió contactar al personal del área de atención médica y del área de presupuesto de los SESA de Oaxaca.

Con respecto al análisis del proceso de teleconsulta, se definió la relevancia de entrevistar al director de alguna unidad médica hospitalaria de mayor productividad para conocer las estrategias que realizan para lograr dichos resultados (cuadro 1).

**CUADRO 1.** Información e instrumentos de recolección, por fuente y tipo de recolección

Acción	Fuentes de información	Instrumentos de recolección	Información recolectada
Trabajo de campo	Coordinador del Programa	Entrevista abierta	Se definió mediante muestreo en cadena a partir de la entrevista con el Coordinador del Programa, quienes serían los actores que podrían aportar información reelevante con relación la institucionalización del programa e impulsar la productividad del programa en los SESA de Oaxaca.
Análisis de gabinete	Coordinación de Telesalud Oaxaca	Documentos y presentaciones electrónicas en formato PDF	Presentaciones de reportes gerenciales de la Coordinación de Telesalud Oaxaca
Análisis de gabinete	Coordinación de Telesalud Oaxaca	Manual de procedimientos del Programa de Telesalud Oaxaca	Organigrama de los SESA Oaxaca
Análisis de gabinete	Coordinación de Telesalud Oaxaca	Acceso al Registro Clínico de Información (Sistema de agenda de teleconsultas)	Manual de usuarios: Registro Clínico de Información, 2010–2016 Estadísticas de productividad de teleconsultas
Análisis de gabinete	Dirección de Telesalud CENETEC-Salud	Documentos y presentaciones electrónicas en formato PDF	Modelo de Atención Médica a Distancia CENETEC-Salud
Trabajo de campo	Médicos generales y especialistas de la Red de Telesalud	Cuestionarios en línea recopilados mediante el *software* de licencia libre Qualtrics^®^	De las 19 unidades de la Red de Telesalud, 16 médicos generales que laboran en 13 unidades respondieron el cuestionario
Trabajo de campo	Médicos especialistas de la Red de Telesalud	Cuestionarios en línea recopilados mediante el *software* de licencia libre Qualtrics^®^	De los seis médicos especialistas que proporcionan teleconsulta, todos respondieron los cuestionarios.
Trabajo de campo	Actores clave involucrados con el Programa	Entrevistas presenciales semiestructuradas	Cuatro entrevistas semiestructuradas a actores de interés del Programa: en general, se recuperaron percepciones de los participantes en con respecto a las alternativas de institucionalización del programa y de impulso a la productividad del mismo.

SESA, Sistemas Estatales de Salud; CENETEC, Centro Nacional de Excelencia Tecnológica en Salud.

En la entrevista inicial con el coordinador se recopilaron los manuales operativos disponibles del proceso de teleconsulta. Con base en estos documentos, se desarrolló un cuestionario para su aplicación electrónica a los 16 médicos generales que laboran en las unidades médicas consultantes. Un cuestionario con tópicos similares se realizó para los médicos especialistas. La información de ambos grupos de informantes fue ordenada según las etapas del proceso de teleconsulta para poder identificar barreras en la implementación.

Finalmente, se integraron en un reporte los instrumentos de recopilación de información y se elaboró un documento final del proyecto en extenso, el cual sirvió como fuente de información para la elaboración del presente artículo.

## Consideraciones éticas

El estudio fue evaluado por el Comité de Ética de la OPS, de acuerdo con el Dictamen de Evaluación para Propuestas de Investigación No. PAHO-2015-03-0013. Antes de la realización de las entrevistas, se recabó el consentimiento informado de los participantes; para los cuestionarios recopilados vía electrónica, se obtuvo el consentimiento informado por el mismo medio.

## RESULTADOS

## El proceso de teleconsulta

En el proceso de teleconsulta, la interacción del médico consultante con el especialista comienza con el envío de la solicitud de teleconsulta. El Programa cuenta con un *software* para el intercambio de la información clínica del paciente. Este sistema tiene la finalidad de proteger la privacidad de la información y administrar la agenda de teleconsultas. Sin embargo, cuando los médicos interconsultantes reciben la solicitud de teleconsultas, cuatro de seis de ellos comentaron que, regularmente, no reciben suficiente información sobre los antecedentes clínicos de pacientes en la solicitud de teleconsulta.

En contraparte, se preguntó a los médicos consultantes cuáles eran las limitaciones que habían identificado para el intercambio de información al inicio, desarrollo y posterior a la teleconsulta. Algunas de sus respuestas fueron: 1) la conectividad y la falta de accesorios e impresora, 2) la falta de capacitación de los colegas del hospital y 3) la coincidencia en los tiempos (horario laboral) por parte del médico especialista y la unidad médica solicitante del servicio.

Con relación a la respuesta recibida cuando realizan una solicitud de teleconsulta, 13 de 16 médicos generales opinaron que la programación de citas por parte de médicos especialistas es oportuna. Un total de diez médicos generales, de 16 consultados, han requerido atención por teleconsulta fuera del horario laboral de la Coordinación de Telesalud (en fines de semana y en horario vespertino). Ocho de 16 médicos generales han requerido servicios de especialidades no disponibles en el Programa, por ejemplo: urología, oncología y dermatología (cuadro 2).

**CUADRO 2.** Fortalezas y debilidades del proceso de teleconsulta

Fortalezas	Debilidades
Trece de 16 médicos generales indicaron que recibieron capacitación sobre el proceso de teleconsulta.	Siete de 16 médicos generales respondieron que son el único médico que realiza atención con teleconsultas en su unidad médica.
Nueve médicos generales señalaron que cuentan con apoyo de otros médicos para las actividades de telemedicina.	La lentitud de la conexión a internet dificulta el envío de la solicitud de teleconsultas en opinión de los médicos generales.
Trece de 16 médicos generales consultados opinaron que la programación de citas por parte de médicos especialistas es oportuna.	El uso y recopilación de un formato de consentimiento informado de los pacientes para realizar teleconsultas no fue registrado en las respuestas de médicos generales y especialistas del programa****.
El equipo periférico con mayor disponibilidad reportado por los médicos generales fue el que contaba con de examinación general, electrocardiógrafos, unidad para ultrasonografía.	Cuatro de seis médicos especialistas respondieron que no reciben antecedentes suficientes en la solicitud de teleconsulta, comentaron que la información es parcial o que solo algunas unidades médicas envían información completa.
El Sistema de Registro de Información Clínica es una plataforma que permite el intercambio de información de los pacientes, la administración de la agenda de consultas y permite obtener estadísticas para el monitoreo del programa.	Diez médicos generales, de 16 consultados, han requerido atención por teleconsulta fuera del horario laboral de la Coordinación de telemedicina, en fines de semana y en horario vespertino.
Continuidad de la atención: en el sistema de administración de agenda, 40% de los pacientes del servicio de medicina interna tienen registrada una teleconsulta, y 60% de los pacientes de la misma especialidad, tienen registradas dos o más teleconsultas. De manera similar, en el servicio de ginecología. 56% de las pacientes tienen registrada una consulta y 44% tienen registradas dos y más teleconsultas.	Ocho de 16 médicos generales han requerido servicios de especialidades no disponibles al momento del estudio en el programa como, por ejemplo, de las especialidades de urología, oncología y dermatología. Los médicos generales reportaron escasa disponibilidad de los siguientes equipos periféricos para exploración del paciente durante las teleconsultas: otoscopio digital, cámara midriática para fondo de ojo, laringoscopio digital y analizador portátil de química clínica.

***Fuente:*** elaboración propia con base en los hallazgos de la investigación.

Cuando hay una interrupción de teleconsultas por fallas en la conexión a internet, los médicos indicaron que se comunican por teléfono, mail o servicios de mensajería como *Whatsapp*^*®*^ para programar la sesión en una fecha próxima.

En cuanto a los elementos normativos, se identificó que es necesario desarrollar manuales de procesos que puedan ser utilizados como material de capacitación a los médicos generales que solicitan consultas al Programa.

En las entrevistas realizadas al coordinador del Programa, al personal del centro de salud y al personal del hospital comunitario, se comentó que los participantes en las consultas suelen ser personas jóvenes o familiarizadas con el uso de computadoras. En el Hospital Comunitario 1, se identificó que cuenta con un lugar para estancia de mujeres embarazadas, y existe coordinación con el personal de las unidades médicas de primer nivel en el área geográfica de influencia del hospital, lo que impulsa la demanda de teleconsultas.

## Análisis de cobertura de atención

En el periodo comprendido entre el 6 de mayo de 2014 y el 27 de agosto de 2015, el Programa proporcionó 4 140 teleconsultas a 1 525 pacientes, en 16 unidades médicas de las 19 que integran la Red de Telesalud de Oaxaca. En este período se realizaron un promedio de ocho teleconsultas por día.

El servicio de medicina interna es el de mayor demanda. Los dos médicos especialistas de esta especialidad proporcionaron 82% (n = 3 395) de las teleconsultas del Programa; las consultas del servicio de ginecología representaron 15% del total de teleconsultas. Cuatro unidades médicas consultantes concentraron 75% (n = 3 105) de las teleconsultas, el resto de las teleconsultas fueron proporcionadas en 12 unidades médicas (cuadro 3).

**CUADRO 3.** Teleconsultas por unidad médica en la Red de Telesalud de Oaxaca.

Unidad médica consultante	Número de teleconsultas	Participación
Hospital Comunitario 1	1 005	24,3%
Hospital Comunitario 2	967	23,4%
Hospital Comunitario 3	763	18,4%
Hospital Comunitario 4	355	8,6%
Hospital Comunitario 5	324	7,8%
Hospital Comunitario 6	312	7,5%
Hospital Comunitario 7	162	3,9%
Unidad Médica Móvil 1	91	2,2%
Hospital 1	88	2,1%
Centro de Salud 1	36	0,9%
Centro de Salud 2	25	0,6%
Unidades médicas diversas (5)	12	0,3%
Total	4 140	100,0%

***Fuente:*** elaboración propia con datos de la Coordinación General de Telemedicina. Registro clínico de Información 2014–2015, Servicios de Salud de Oaxaca, Gobierno del Estado de Oaxaca, 2010–2016.

En cuanto al seguimiento de los pacientes, se identificó que 40% de los pacientes del servicio de medicina interna (n = 450) recibieron una consulta en el período de estudio y 60% (n = 677) recibieron dos o más teleconsultas. De manera similar, en el servicio de ginecología, 56% (n = 186) de las pacientes recibió una consulta y 44% (n = 145) dos y más teleconsultas. Lo anterior concuerda con las opiniones de los médicos sobre la utilidad del uso del servicio de telemedicina.

## Cambios posteriores a la investigación de implementación

Después de la difusión de resultados de la investigación en los SESA de Oaxaca, se registraron los siguientes cambios en el Programa que favorecen su institucionalización:

- El 24 de noviembre de 2015 la Coordinación de Telesalud fue integrada a la estrategia de fortalecimiento de las redes de unidades médicas del estado. La coordinación supervisará las referencias de pacientes realizadas por unidades médicas consultantes a fin de disminuir la saturación de hospitales.
- El 15 de enero de 2016 dicha Coordinación en Oaxaca fue reconocida en el Reglamento Interno de los Servicios de Salud de Oaxaca. Esto representa un avance en la formalización y establecimiento del Programa en la organización.
- A partir del 9 de enero de 2016, se amplió el horario de atención de los médicos especialistas del servicio de medicina interna, ahora estará disponible los días sábado, domingo y días festivos.

## DISCUSIÓN

La Estrategia Nacional de Telesalud en México depende del impulso en términos de recursos humanos y materiales que cada institución proporcione a sus proyectos. En este supuesto se encuentran los programas de telesalud como el de Oaxaca y el resto de los programas al interior de los SESA. Cabe destacar que la etiqueta de “programa” no implica que la organización de los servicios de telemedicina cuente con un presupuesto predestinado para su operación.

Los principales desafíos de los programas de telesalud son las políticas, la infraestructura y la capacitación del recurso humano.

En general, los SESA operan en un entorno de escasez relativa de médicos e insumos para la atención. En este sentido, puede deducirse que la dedicación de los médicos al Programa de telesalud implica un intercambio de tiempo (costo de oportunidad) entre la atención persona a persona y la atención vía telemedicina, en especial en el caso de los médicos generales en zonas remotas (9). Los SESA tienen el desafío de cubrir la atención en zonas marginadas, aisladas y dispersas, ya que en estas zonas se encuentran, principalmente, los beneficiarios potenciales de los programas como el de Telesalud de Oaxaca. Para la población con mayor desigualdad, la atención médica que proporciona el Programa de Telesalud tiene el potencial de evitar gastos de transporte. Este ahorro en gastos de transporte es relevante si se toma en cuenta que el gasto de bolsillo en México, en 2014, representó 52% del gasto en salud (23).

En congruencia con el método iPier, existen varias recomendaciones en la literatura sobre el uso de metodologías de evaluación en telemedicina que sugieren el uso de evaluaciones formativas, las cuales están emergiendo como un área de interés en la investigación del campo, así como la aplicación de diferentes enfoques naturalistas, que incluyen metodologías que abordan adaptaciones mutuas de servicios y usuarios (24–26).

Ante la concentración de la productividad en unidades médicas y en la especialidad de medicina interna, puede considerarse que la maduración del Programa está igualmente concentrada o es dispar. Por el momento, no se recomienda avanzar en la búsqueda de estudios de costo-efectividad, sino dirigir los esfuerzos del Programa para definir protocolos de atención y catálogos de diagnósticos atendibles mediante la consulta de telemedicina.

Es recomendable utilizar la metodología de marco lógico (27) para armonizar propuestas de indicadores de resultado en términos de productividad, reducción de tiempos de atención o ahorro en costos para pacientes (28). Cabe precisar que estos indicadores requieren un periodo de prueba para identificar cuáles son de mayor utilidad para la toma de decisiones de los gerentes del Programa. En cambio, para estudiar el seguimiento la atención médica de los pacientes en forma global, es suficiente considerar indicadores de atención de servicios de salud como la clasificación de consultas de primera vez y subsecuentes.

En cuanto a las limitaciones del estudio, cabe mencionar que existen dificultades geográficas para hacer visitas presenciales a las unidades médicas rurales consultantes del Programa. Durante la ejecución del estudio, el sindicato de trabajadores de los SESA de Oaxaca entró en huelga, lo cual fue una dificultad para el desarrollo del estudio; aunque, en realidad, se trata de una dificultad más grave para la prestación del servicio.

Respecto a estudios basados por completo en métodos cualitativos (29), el muestreo cualitativo realizado en este estudio fue reducido, por las limitantes geográficas y la coyuntura de operación de los SESA en Oaxaca. Sin embargo, el método y la muestra empleados fueron suficientes para encontrar saturación en los temas de interés del estudio. Asimismo, se encontró coincidencia con la información recopilada mediante las entrevistas, las encuestas y la información cuantitativa.

Un aspecto a destacar en la investigación fue la recopilación de información de médicos generales en unidades médicas rurales mediante el cuestionario suministrado en línea. El uso de este medio permitió obtener respuestas con mayor privacidad, en comparación con realizar una visita presencial para aplicar el cuestionario. Se encuentra pendiente indagar el conocimiento de la población acerca de la disponibilidad de teleconsultas en las unidades médicas y si este conocimiento pudiera impulsar la productividad.

Se observó que las barreras en la implementación del proceso de teleconsulta las tienen, principalmente, los médicos generales de las unidades consultantes. El diseño de programas de telesalud enfatiza las condiciones de la oferta del servicio, la adquisición de equipo, la conectividad, y la disponibilidad de médicos interconsultantes. Para contrarrestar esta deficiencia, se requiere estimar la disponibilidad de los médicos generales o consultantes. De ellos depende el número de solicitud de teleconsultas.

La coordinación de los equipos de atención primaria a la salud con el hospital comunitario permite incrementar la productividad del Programa, en sincronía con el impulso del director de la unidad médica consultante para facilitar las condiciones materiales y el mantenimiento de la tecnología de comunicación.

## Conclusión

En el entorno bajo el que opera el Programa de Telesalud de Oaxaca se encuentran los pacientes que mayor dificultad de acceso tienen a servicios de salud de especialidad.

En cuanto a la oferta de atención del Programa, es una ventaja que los médicos especialistas laboren un turno dedicados exclusivamente a la atención por telemedicina. Es deseable que el Programa cuente con comunicación con los hospitales generales adonde se referencia a los pacientes que así lo requieren, a fin facilitar el acceso a la atención y contribuir a la incorporación del Programa a la red de servicios.

En contraparte, la demanda de atención del Programa tiene obstáculos como la participación de los médicos generales — en un entorno de escasez de médicos—, la conexión a internet, la disponibilidad de insumos y mobiliario para realizar las teleconsultas.

Establecer estrategias de monitoreo del Programa de Telesalud tiene el potencial de proveer información para difundir con los tomadores de decisión los beneficios que tiene el programa para pedir mayores apoyos para el desarrollo del entorno tecnológico y operativo en el que actúa el programa para contribuir a reducir las inequidades en salud.

## Agradecimientos.

Los autores agradecen a Marcelo D’Agostino (OPS), a Etienne Vincent Langlois (AHPSR), a Ludovic Reveiz (OPS) y a Gabriel Sainz Coronado (Programa de Telesalud) por su colaboración en la elaboración de este manuscrito.

## Financiamiento.

Este trabajo fue financiado por la Alianza para la Investigación en Políticas y Sistemas de Salud (AHPSR) de la Organización Mundial de la Salud (OMS) y la Organización Panamericana de la Salud (OPS). La OPS brindó cooperación técnica para el desarrollo del proyecto. En el contexto del programa iPIER, el Instituto de Efectividad Clínica y Sanitaria (IECS) brindó asistencia técnica para el desarrollo del protocolo y la ejecución del proyecto.

## Declaración.

Las opiniones expresadas en este manuscrito son responsabilidad del autor y no reflejan necesariamente los criterios ni la política de la *RPSP/PAJPH* y/o de la OPS/OMS.

## Footnotes

2 De acuerdo con el Modelo Integrador de Atención a la Salud MIDAS (2006), el hospital de la comunidad representa el espacio de salud donde se resuelve gran parte de los problemas de salud que requieren internación. Su relación dentro del sistema de redes garantizaría fácil acceso a un hospital con mayor capacidad resolutiva para canalizar a los pacientes que así lo requieran. El tiempo de recorrido para los pacientes o usuarios de la población debería ser no mayores de 60 minutos y la distancia por recorrer máxima de 15 kilómetros. Su capacidad promedio es de 12 a 18 camas hospitalarias. Secretaría de Salud de México. Dirección General de Planeación y Desarrollo en Salud. Planeación de Unidades Médicas: Modelo Integrador de Atención a la Salud MIDAS. Primera edición, 2006.
